# Tailoring pharmacotherapy to specific eating behaviours in obesity: Can recommendations for personalised therapy be made from the current data?

**DOI:** 10.1007/s00592-017-0994-x

**Published:** 2017-04-19

**Authors:** Carl A. Roberts, Paul Christiansen, Jason C. G. Halford

**Affiliations:** Department of Psychological Sciences, Institute of Psychology, Health and Society, University of Liverpool, Eleanor Rathbone Building, Bedford Street South, Liverpool, L69 7ZA UK

**Keywords:** Pharmacotherapy, Obesity, Eating behaviour, Appetite, Reward, Inhibitory control, Personalised Medicine

## Abstract

Pharmacotherapy provides an adjunct to behaviour modification in the management of obesity. There are a number of new drug therapies purportedly targeting appetite; liraglutide, and bupropion/naltrexone, which are European Medicines Agency and US Food and Drug Administration (FDA) approved, and lorcaserin and phentermine/topiramate, which have FDA approval only. Each of the six drugs, used singly or in combination, has distinct pharmacological, and presumably distinct behavioural, mechanisms of action, thus the potential to provide defined therapeutic options to personalise the management of obesity. Yet, with regard to pharmacotherapy for obesity, we are far from true personalised medicine. We review the limited mechanistic data with four mono and combination pharmacotherapies, to assess the potential for tailoring their use to target specific obesogenic behaviours. Potential treatment options are considered, but in the absence of adequate research in respect to effects of these drugs on eating behaviour, neural activity and psychological substrates that underlie poorly controlled eating, we are far from definitive therapeutic recommendations. Specific mechanistic studies and broader behavioural phenotyping, possibly in conjunction with pharmacogenetic research, are required to characterise responders for distinct pharmacotherapeutic options.

## Introduction

The global obesity pandemic is a primary public health concern, due to prevalence (600 million obese, BMI ≥ 30, within a wider population of 1.9 billion overweight adults according to WHO), and the impact excess body weight has on physical, psychological and economic quality of life [1]. In the UK, the annual cost of obesity to the NHS is an estimated £5.1 billion, whilst total cost to the wider economy is an estimated £27 billion [2]. Therefore, effective measures to tackle the burden of obesity, and obesity related diseases, are essential.

Reducing energy intake through changes in eating behaviour, and increasing daily activity, help maintain the state of negative energy balance required to lose weight. These are the principle components of obesity treatment and demand fundamental and sustained behavioural change. In the context of intervention, there are two key barriers to behaviour change; firstly, patterns of eating and activity behaviour are shaped by lifelong learning, and behaviour modification must tackle entrenched habit. Secondly, even if change is achieved, maintaining healthier behaviours in an environment that promotes weight gain demands constant exertion. The obesogenic environment primes individuals to relapse into their pre-intervention behavioural repertoire. Problematically, appetite regulation is asymmetrical [3], in that the body defends against under-consumption irrespective of current weight status or energy reserves.

Appetite regulation involves an interplay between satiety, inhibitory control (IC) and reward processes. The obese have a biological vulnerability for weight gain which is manifested in eating behaviours that lead to overconsumption [4]. Blundell et al. [5] suggested a cluster of behaviours that relate to satiety (weak satiety response, weakened post-ingestive satiety), reward (preference for high fat foods, strong hedonic attraction to palatable foods), and IC (disinhibited eating, uncontrolled hunger) that comprise a susceptible behavioural phenotype for obesity. Thus, regulatory control of eating is undermined by reduced satiety and increased responsivity to food cues (reward driven eating). As such, IC has greater likelihood of being overwhelmed by environmental cues to over consume in the obese.

Calorie restriction compromises appetite control by increasing responsivity to food cues (an effect which is even more pronounced in overweight/obese individuals) [6], craving [7] and preoccupation with food [8], as well as having negative impact on mood and cognition [9]. Negative mood states can lead to reduced control of eating and increased emotional eating [10]. Taken together, this suggests that the obese are arguably the least capable of coping with the consequences of dieting, as their appetite is prior compromised, they have low control over their eating, and often suffer with depression, impacting upon motivation.

Heritability estimates of body weight are high. However, multiple common genetic variants belie obesity in the general population. Recent meta-analyses suggest that 97 BMI-associated genetic loci account for under 3% of variation in BMI [11], thus the variance of BMI explained by any single gene is low. This suggests that a standardised personalised medicine approach of targeting key genes with pharmacotherapy for obesity would, on its own, be inadequate. However, the fat mass and obesity-associated gene (FTO) has the largest effect size of BMI-associated genetic variants, whereby adults homozygous for the at risk allele are approximately 3 kg heavier than those not inheriting the at risk allele [12]. Notably, Wardle et al. [13] found that variation in FTO is associated with diminished satiety, and more recently satiety sensitivity was shown to mediate part of the association between genetic risk and adiposity [14]. Similarly variations in FTO are associated with other adiposity-related behaviours in children; increased food intake [15] fat consumption/consumption of palatable food [16] and loss of control over eating [17]. Recent research in dizygotic twins from the GEMINI population-based twin cohort suggests that appetite differences in the first few weeks of life yield differential weight gain from 3 to 15 months [18], and meal size is an important driver of weight gain in early life [19]. Taken together, these findings suggest that differences in behaviour are critical in mediating the association between genetic risk and obesity.

In order to personalise pharmacotherapy for obesity in adults, it is critical that behavioural issues associated with obesity are targeted. How does drug therapy impact appetite (within a meal and throughout the day), portion size and the frequency of eating (satiety)? How does it affect responsiveness to palatable foods, eating rate and food choice (reward)? Can pharmacotherapy improve the ability to control eating behaviour (IC)? If so, can treatment be tailored to patients with specific problem behaviours?

## Personalised therapy

**Fig. 1:**
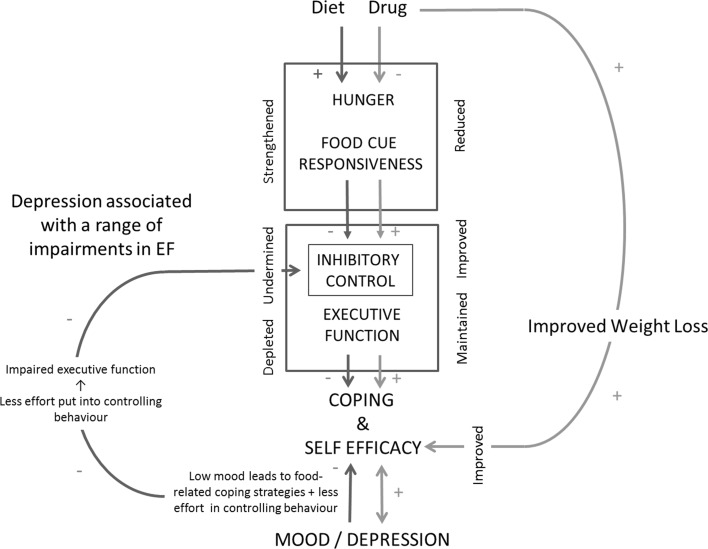
Mitigating effects of pharmacotherapy on calorie restriction and negative sequelae. Dieting increases hunger and food cue responsiveness, which undermines inhibitory control and other executive functions and in turn, the ability to cope and maintain the diet (self-efficacy). Dieting also has negative effects on mood which reduce self-efficacy for controlling behaviour and reintroduce food-related coping strategies. Negative mood state also reduces inhibitory control and other executive functions producing a cycle whereby ability to control behaviour and self-efficacy is undermined. However, anti-obesity drugs can mitigate some of the effects of dieting by reducing hunger and food cue responsiveness, leading to improved inhibitory control and maintained executive function, which improves self-efficacy and the ability to maintain calorie restriction. Mitigating effects on mood are also boosted by improved coping and self-efficacy, as well as by observing improved weight loss

Specialised adult weight management services provide person-centred care, for treatment of obesity, assisting patients to make lasting lifestyle changes. In this setting, access to distinct pharmacotherapies could well provide meaningful benefits when developing individual management plans. Recent NICE-accredited commissioning guidance for weight management clinics recommends a specialist multidisciplinary team approach (including pharmacotherapy) for treatment of severe obesity [20]. Pharmacotherapy has the potential to improve weight loss outcomes in primary care and specialised weight management services, through (1) reducing negative psychological and biological sequelae produced by calorie restriction, (2) aiding behaviour change and (3) improving self-efficacy (Fig. 1). However, the development of a personalised approach is not possible without guidance from patient experience about personal barriers to behaviour change. In addition, pharmacogenetic and mechanistic studies, which identify drug effects on eating behaviour, IC, and reward processing, would allow characterisation of successful responders to different treatments.

Here, we review the available data assessing behaviour and neurophysiology modification with the four most recently available (FDA approved) mono and combination pharmacotherapies for obesity. Whilst orlistat (Xenical) has been approved as a pharmacological intervention for obesity since 1998, this is not a centrally acting drug and is not regarded as having direct behavioural effects (although its side effect profile may modify food choice and encourage adherence to a low fat diet) relating to satiety, inhibitory control or reward responsivity, for these reasons it has not been included in this review.

## Liraglutide (Saxenda)

Liraglutide is a glucagon-like peptide-1 (GLP-1) receptor agonist with long-lasting biological activities (half-life of 10–14 h) in comparison with endogenous GLP-1 which is rapidly degraded by the enzyme dipeptidyl peptidase-IV (DPP-IV). Liraglutide is approved for treatment of T2DM at the 1.8 mg dose, and recently attained FDA (2014) and EMA (2015) approval for use as a weight loss therapy at the 3.0 mg injectable dose.

### Mechanism of action

Endogenous GLP-1 is secreted from L endocrine cells in the intestine in response to intraluminal nutrients and stimulates GLP-1 receptors located on pancreatic beta cells, to promote glucose-dependent insulin secretion [21]. GLP-1 is therefore recognised as an incretin hormone. GLP-1 is also synthesised in the nucleus tractus solitarius (NTS) of the brainstem and acts as a neurotransmitter projecting to feeding relevant hindbrain, midbrain and forebrain regions. The central actions of gut-derived and brain-derived GLP-1 may be separable. Due to the rapid degradation of gut-derived GLP-1 by DPP-IV, GLP-1 contributes a neuroincretin effect via a neural pathway composed of vagal afferents in the intestine and hepatic portal, and not by crossing the blood brain barrier (BBB) [22]. Knockdown of vagal afferent neuron GLP-1Rs increases meal size and accelerates gastric emptying, as well as producing elevated post-meal glycaemia and reduced insulin, highlighting the neuroincretin and satiety effects of gut-derived GLP-1 [23].

Long-lasting GLP-1R agonists, such as liraglutide, are DPP-IV resistant, remain stable following peripheral administration and can have central effects from crossing the BBB as well as via vagal afferents. As such, liraglutide exhibits pleiotropic effects which extend beyond the incretin action of gut-derived GLP-1 [22], via action at central GLP-1R. This is supported by pre-clinical evidence that vagotomy only partially blocks intake suppression following intraperitoneal liraglutide injection [24].

Peripherally administered liraglutide has been observed to act directly on POMC neurons in the arcuate nucleus of the hypothalamus [25], to suppress feeding. Moreover, central GLP-1Rs are also located in the mesolimbic reward system, e.g. the ventral tegmental area (VTA), and nucleus accumbens [26]. Thus, liraglutide may also influence reward-motivated eating and reduce intake through effects on central appetite and reward neural pathways, as well as peripheral gastrointestinal sites.

### Efficacy, effects on behaviour and tailoring potential

Human data on the efficacy of liraglutide for weight loss come from the Satiety and Clinical Adiposity–Liraglutide Evidence (SCALE) studies. These studies suggest liraglutide 3.0 mg is effective at reversing prediabetes and producing weight loss. A meta-analysis of three studies, with a total of 2921 patients receiving liraglutide and 1503 receiving placebo, suggests 5.2 kg additional weight loss and an OR of 5.54 for achieving 5% weight loss with liraglutide relative to placebo [27].

Short-term liraglutide treatment (20.0 ± 6.4 days) at low doses (0.3–0.9 mg per day) can reduce staple food intake (but not non-staple food intake) and feelings of hunger, compared to other oral glucose lowering medication [28]. Reductions in hunger along with reduced duration of eating during an ad libitum buffet were reported with liraglutide (1.8 mg dose), relative to placebo and glimepiride (oral sulfonylurea used as an active control to discriminate appetite and glycaemic control effects) in overweight/obese (BMI 27–40 kg/m^2^) males and females with T2DM [29]. Nevertheless, there was no reduction in total energy nor macronutrient intake. However, an 18% reduction in energy intake at an ad libitum buffet lunch meal, accompanied by lower postprandial hunger, with liraglutide (1.8 mg) compared to placebo, was reported in another sample of males and females (BMI 29.7  ±  4.2 kg/m^2^) with T2DM. No change in macronutrient composition or meal duration was seen [30]. Taken together, these data suggest that even at sub-therapeutic doses for weight management, there is consistent evidence of drug effects on satiety in a population generally overweight or obese, albeit all with T2DM.

Using both 3.0 and 1.8 mg doses in non-diabetic adults, van Can et al. [31] observed that both doses produced ~16% reduction in food intake in an ad libitum lunch meal relative to placebo (5 h post-fixed load breakfast). Post-meal satiety and fullness ratings were significantly increased in both liraglutide groups versus placebo, and prospective consumption was significantly reduced. The 3.0 mg dose also delayed gastric emptying 1 h post-breakfast compared to placebo. Twenty-four hour energy expenditure was reduced in liraglutide treatment groups relative to the control; this pattern of results suggests that weight loss with liraglutide is produced by reductions in food intake and increased satiety rather than changes in energy expenditure.

With regard to central mechanisms, Farr et al. [32] reported decreased parietal activation in response to rewarding food images following 17 days of liraglutide treatment (0.6 mg for 7 days, 1.2 mg for 7 days and 1.8 mg for 3 days) versus placebo, in obese patients with T2DM, tested in the fasted state. It was argued this reflects an attenuation of appeal of energy dense foods. Moreover, reduced parietal activation to palatable foods was correlated with ratings of how pleasant participants would find eating. However, the sample size was small (*n* = 18) so findings should be treated with caution (see [33]). IC was assessed (out of the scanner using Stop Signal and Go/No-Go Tasks), but no effects of liraglutide were found. However, during the fasted state, liraglutide significantly reduced hunger ratings relative to placebo, and daily energy intake was significantly reduced at 1.8 mg liraglutide vs placebo (although how this was assessed is not reported).

Liraglutide, therefore, appears to produce weight loss through homoeostatic mechanisms that boost satiety, leading to reduced intake. However, more data are needed with the 3.0 mg dose in non-T2DM samples to characterise effects on the interplay between satiety, reward and IC.

## Bupropion/Naltrexone (Contrave)

The combination therapy of the catecholamine reuptake inhibitor bupropion (360 mg/day) and opioid antagonist naltrexone (32 mg/day) (Table 1) is approved for the management of obesity in sustained release form by the FDA and the EMA. Bupropion is an atypical antidepressant, which is currently used in smoking cessation to reduce craving and ease withdrawal [34]. In addition, bupropion also has an observable anorexigenic effect. Clinical trials investigating bupropion as a monotherapy demonstrate modest weight loss [35, 36]. Naltrexone has also been investigated as monotherapy for obesity, but although it affects food choice and palatability, it is not associated with weight loss [37]. Nevertheless, naltrexone’s potential to reduce reward sensitivity to palatable food may be beneficial in a combined therapy.

**Table 1:** Mechanism of action and effect on appetite expression, eating behaviour and CNS activity for weight loss pharmacotherapies

Drug	Mechanism of action	Effect on appetite expression, eating behaviour or CNS activity
Liraglutide	GLP-1 receptor agonist	Reduced intake, reduced post-meal hunger, increased post-meal satiety and fullness. Reduced CNS reward activity
Bupropion/naltrexone	Dopamine and norepinephrine reuptake inhibitor+opioid (mu and k) receptor antagonist	Increased fullness, reduced hunger, reduced desire for sweet, non-sweet or starchy foods, increased ability to control eating and resist craving. Increased activity in inhibitory control-related areas (anterior cingulate), reduced activity in hypothalamus
Lorcaserin	Selective 5HT_2C_ receptor agonist	Reduced food intake, decreased hunger, decreased activity in attention-related neural regions (parietal and visual cortices), reduced emotional and salience related limbic activity (insula and amygdala)
Phentermine/topiramate	TAAR1 agonist and norepinephrine releasing agent+sulphamate-substituted monosaccharide with action on GABA signalling	No published data

### Mechanism of action

Pre-clinical studies demonstrate the bupropion/naltrexone combination acts on POMC neurons, critical components in the integration of episodic and tonic signals of energy intake and energy storage (expressing both serotonin and leptin receptors). Bupropion stimulates the release of the MC_4_R agonist αMSH from the POMC neurons in the PVN. The POMC neuron itself is regulated by endogenous opioids via opioid-mediated negative feedback. Naltrexone blocks the inhibitory action of β-endorphin preventing the cessation of bupropion-induced activation [38]. Due to the key role of POMC neurons in integrating and relaying peripheral satiety signals, it is hypothesised bupropion/naltrexone reduces hunger by strengthening within-meal satiation and post-meal satiety.

### Efficacy, effects on behaviour and tailoring potential

Efficacy of bupropion/naltrexone to produce weight loss in overweight or obese adults has been assessed in the Contrave Obesity Research (COR) phase III clinical trials [ [39]–[41]—see Table 2]. Meta-analysis suggests 5.0 kg additional weight loss and an OR of 3.96 for achieving 5% weight loss with bupropion/naltrexone relative to placebo (*n* = 2044 vs. 1319 over four studies) [27].

**Table 2:** Mechanistic studies included in review

Authors and year	Drug	Participants	Appetite effects/CNS activity
Inoue et al. 2011	Liraglutide 0.9 mg compared to oral glucose lowering medication	*n* = 20 (12 female), T2DM	Decreased daily staple food intake, but not intake for non-staple food. Decreased external eating, reduced preference for fatty foods
Horrowitz et al. 2012	Liraglutide 1.8 mg, glimepiride and placebo, incomplete Latin square design	*n* = 46 (19 female), T2DM, BMI 27–40 kg/m^2^	Decreased fasting hunger, shorter meal duration. No significant effects on intake
Flint et al. 2013	Liraglutide 1.8 mg—randomised, placebo-controlled, double-blind, crossover design	*n* = 18 (4 female), T2DM, BMI 18.5–40 kg/m^2^	After 3 weeks of treatment, liraglutide decreased energy intake by 18% at *ad lib* buffet meal, no difference in macro nutrient intake or meal duration. Decreased mean 5 h postprandial hunger ratings
van Can et al. 2014	Liraglutide 1.8 mg, 3 mg or placebo—randomised placebo-controlled, double-blind, incomplete crossover trial	*n* = 49 (20 female), obese, BMI 30–40 kg/m^2^	Decreased *ad lib* food intake (lasagne), increased satiety and fullness (post-meal), reduced prospective consumption (post-breakfast)
Farr, et al. 2016a	Liraglutide 1.8 mg—randomised, placebo-controlled, double-blind, crossover trial.	*n* = 18 (9 female) T2DM, placebo BMI: 31.2 ± 1.7 placebo, liraglutide BMI: 31.91 ± 1.7†	Decreased activation in insula and putamen (reward areas) whilst viewing food pictures. No between-group differences in neurocognitive measures (including inhibitory control SSRT and Go/No-Go)
Greenway et al. 2010	NB32 or NB16—randomised, placebo-controlled, double-blind, trial	*n* = 583 (496 female) NB32, *n* = 578 (490 female) NB16, *n* = 581 (496 female) placebo. Obese, BMI 30–45 kg/m^2^, without complications or BMI 27–45 kg/m^2^ with controlled hypertension, dyslipidaemia or both	NB32 and NB16 increased control of eating and ability to resist cravings, reduced hunger, desire for sweet, non-sweet or starchy foods, increased fullness. No drug effects on FCI score or subscales
Wadden et al. 2011	NB32—randomised, placebo-controlled, double-blind trial	*n* = 202 (185 female) placebo plus behaviour modification, *n* = 591, 89.3% female (NB32 plus behaviour modification) obese BMI 30–45 kg/m^2^, without complications or BMI 27–45 kg/m^2^ with controlled hypertension, dyslipidaemia or both	Increased control of eating on COEQ, no other differences (cravings, appetite, eating behaviour). No significant differences on FCI
Apovian et al. 2013	NB32—randomised, placebo-controlled, double-blind trial	*n* = 1001 (847 female) NB32, *n* = 495 (419 female) placebo. Obese BMI 30–45 kg/m^2^, without complications or BMI 27–45 kg/m^2^ with controlled hypertension, dyslipidaemia or both	Increased ability to resist cravings and control eating, reduced frequency of food craving
Wang et al. 2014	NB32—randomised, placebo-controlled, double-blind crossover trial	*n* = 40 (all female), overweight and obese BMI 27–45 kg/m^2^	Decreased activation in hypothalamus (increased satiety), increased activation in anterior cingulate (inhibitory control), superior frontal, insula, superior parietal (internal awareness) and hippocampus (memory) relative to placebo
Martin et al. 2010	Lorcaserin (10 mg twice daily)—randomised, placebo-controlled, double-blind trial	*n* = 29 (20 female) lorcaserin, *n* = 28 (19 female) placebo, overweight and obese BMI 27–45	Reduced energy intake at *ad lib* lunch and dinner, reduced appetite and hunger
Farr et al. 2016b	Lorcaserin (10 mg twice daily)—randomised, placebo-controlled, double-blind crossover trial	*n* = 48 (25 female), obese. Placebo BMI: 34.2 ± 1.2 placebo, lorcaserin BMI 40.4 ± 1.3†	Decreased activation, compared to baseline in attention-related parietal and visual cortices in response to highly palatable food at 1 week (fasted), and parietal activation in response to any food cue at week 4 (fasted). Decreased limbic activity (insula, amygdala) at baseline predicts weight loss. Reduced energy intake. No between-group differences in neurocognitive testing (including inhibitory control SSRT)

*T2DM* type 2 diabetes mellitus, *BMI* body mass index (kg/m^2^), *GIP* gastric inhibitory peptide, *DLPFC* dorsolateral prefrontal cortex, *COEQ* control of eating questionnaire, *FCI* food craving inventory, *NB32* 360 mg bupropion plus 32 mg naltrexone, *NB16* 360 mg bupropion plus 16 mg naltrexone, *SSRT* Stop Signal Reaction Time

† Mean BMI as range not given

Appetitive traits were assessed using self-report measures in these trials. In COR-I [39] bupropion/naltrexone produced improvements on selected items in the Control of Eating Questionnaire (COEQ), namely increased fullness, reduced hunger (satiety effects), reduced desire for sweet, non-sweet or starchy foods (reward effects), increased ability to control eating and resist food cravings (craving control). Similarly naltrexone/bupropion-treated patients in COR-II [40] report increased ability to resist cravings and control eating, as well as reduced frequency of food craving (as measured by COEQ) again suggesting effects on reward and control (if not satiety) at week 56. No differences in cravings were found on any subscale of the Food Craving Inventory (FCI) between bupropion/naltrexone and placebo in COR-I and COR-BMOD. It is likely that these inconsistencies are the result of the properties of the scales. Specifically the items COEQ relating to craving refer to the ability to control/resist cravings, whereas the items on the FCI relate more to changes in cravings (i.e. preferences for specific foods) perhaps reflecting that this drug combination may not have strong effects on food choice. Indeed, this highlights the importance of clearly operationalising craving assessments from the outset of trials. Overall, across these studies self-report data suggest effects on satiety, reward and/or craving control; however, appropriate direct assessments of drug effects on appetite and behaviour are lacking.

There are no clinical experimental data available for the effects of bupropion/naltrexone effects on eating behaviour. However, fMRI data suggest the combination therapy may be useful for improving control of eating [42]. Specifically, bupropion/naltrexone reduced hypothalamic (satiety) activity and increased anterior cingulate activity (IC) in response to food cues. However, without behavioural correlates and further mechanistic data, the suggestion of improved control remains speculative.

## Lorcaserin (Belviq)

The selective 5HT2C receptor agonist lorcaserin is approved in oral form in the USA (FDA) for long-term weight management in the obese and overweight. Under the trade name Belviq (Arena Pharmaceuticals, San Diego, CA) it is available at the 10 mg BID dose and as Belviq XR at the 20 mg QD dose.

### Mechanism of action

Lorcaserin is understood to reduce food intake by its activation of 5-HT via POMC neurons, importantly this drug is selective for the 5-HT2C receptor, with little affinity for the 2A and 2B receptor subtypes. This limits the psychological and cardiovascular side effects observed with non-selective serotonergic drugs previously approved, then withdrawn, e.g. fenfluramine and sibutramine [43]. Stimulation of 5-HT2C receptors leads to stimulation of POMC in the arcuate nucleus, and αMSH is produced from POMC neurons and acts on MC_4_R which results in food intake reduction in pre-clinical studies [44].

### Efficacy, effects on behaviour and tailoring potential

Weight loss with lorcaserin has been assessed in the Behavioural Modification and Lorcaserin for Overweight and Obesity Management (BLOOM; 3182 obese or overweight patients receiving lorcaserin 10 mg, or placebo, twice daily–Smith et al. 2010) [45], BLOOM-DM (604 overweight or obese T2DM patients receiving lorcaserin 10 mg once daily, twice daily or placebo) [46] and Behavioural Modification and Lorcaserin Second Study for Obesity Management (BLOSSOM; 4008 obese or overweight patients receiving 10 mg once daily, twice daily or placebo) [47] clinical trials. Meta-analysis suggests 3.2 kg additional weight loss and an OR of 3.10 for achieving 5% weight loss with lorcaserin relative to placebo (*n* = 3350 vs. 3288 over three studies) [27].

Martin et al. [48] assessed energy intake and expenditure in overweight/obese participants, following lorcaserin treatment over a 56-day period, in a double-blind, randomised, placebo-controlled trail. At 56 days lorcaserin treatment resulted in significantly reduced energy intake at lunch and dinner in ad libitum buffet meals compared to baseline measures, as well as a significantly larger reduction in energy intake compared to placebo. Reductions in energy intake correlated with reductions in body weight. However, energy expenditure, substrate oxidation and activity levels were not affected by lorcaserin, suggesting that weight loss occurs through reduced energy intake alone. Notably, lorcaserin produced significant decreases in perceived hunger, with no effect on food cravings, body image, dietary restraint and disinhibition.

Lorcaserin effects on BOLD response to food cues have been assessed in a randomised, placebo-controlled trial [49]. Participants were assigned to lorcaserin or placebo conditions and undertook fMRI scans at baseline (pre-drug), one week, two weeks and four weeks in both fasted and fed states. At baseline, there were no between-group differences in neuronal activation. Following one week of treatment the lorcaserin group showed reduced activity in attention-related parietal and visual cortices, to highly desirable food cues, compared to baseline in the fasted state (although this effect was attenuated by week four). Nevertheless, in the fed state, compared to baseline, four weeks of lorcaserin treatment, led to reductions in parietal cortex activation, for all food images relative to non-food images—accompanied by modest weight loss. The authors argue that highly palatable foods become less important to patients receiving lorcaserin due to reduced activation in areas associated with attention and salience. IC was assessed using a Stop Signal Task, with no between-group differences, nor any within group differences over time. A whole-brain analysis of baseline activity suggested that amygdala activation in response to highly palatable food cues predicted greater weight loss at four weeks with lorcaserin. Similarly, baseline amygdala and occipital activation to highly palatable food in a fed state predicted reduced BMI with lorcaserin after four weeks. Thus, lorcaserin may be beneficial to patients who have high food cue reactivity, or are emotional eaters; however, without confirmatory subjective and behavioural data this remains speculative.

More data are needed to fully characterise lorcaserin effects on eating behaviour. However, these two studies tentatively suggest lorcaserin has a modest effect on appetite leading to reduced energy intake and perceived hunger, as well as reduced salience of rewarding food cues. However, there are no behavioural data available to assess impact on reward-motivated, or poorly controlled eating. Whilst IC has been assessed experimentally, with no evidence that lorcaserin increases control, the task used does not appear to be food cue specific, which may provide a more reliable outcome.

## Phentermine/Topiramate (Qsymia)

Phentermine/topiramate extended-release is another combination therapy that is FDA approved for weight management in the obese and overweight with one or more weight related comorbidity (initial dose: phentermine 3.75 mg/topiramate 23 mg extended-release, maintenance dose: phentermine 7.5 mg/topiramate 46 mg extended-release). Phentermine is currently approved singly for short-term obesity management, and topiramate is used in the treatment of epilepsy and migraine prevention [50]. The combination of phentermine/topiramate as a pharmacotherapy for obesity is understood to combine lower doses of each drug to reduce undesired cardiovascular effects whilst having a synergistic effect on weight loss.

### Mechanism of action

The atypical amphetamine derivative phentermine stimulates norepinephrine release in the CNS, with limited effects on dopamine and serotonin [51]. Its action on norepinephrine is understood to produce its anorectic effects. A meta-analysis [52] suggests that monotherapy phentermine produces modest weight loss for up to 6 months. However, despite the absence of addictive potential, due to its status as an amphetamine derivative, phentermine is presumed to increase blood pressure and heart rate (although there is evidence to contrary see Hendricks et al. [53]) and thus is approved for short-term treatment only. Topiramate is a sulphamate-substituted monosaccharide that is widely available as an anti-convulsant, with weight loss properties. The exact mechanism of action of topiramate for weight loss is unclear; nevertheless, dose-ranging studies have observed significant weight loss over 6 months in the obese [54], as well as significant weight loss at 60 weeks with 96, 192 and 256 mg/day (7, 9.1 and 9.7%, respectively) [55].

### Efficacy, effects on behaviour and tailoring potential

Efficacy and safety of two doses of phentermine/topiramate were assessed in the phase III clinical trials CONQUER (2487 overweight or obese patients receiving placebo, once-daily phentermine 7.5 mg/topiramate 46.0 mg, or once-daily phentermine 15.0 mg/topiramate 92.0 mg) [56], SEQUEL (866 patients in continuation study from CONQUER) [57] and EQUIP (1267 obese patients receiving placebo, phentermine 3.75 mg/topiramate 23 mg extended or phentermine 15 mg/topiramate 92 mg) [50]. In a meta-analysis [27] phentermine/topiramate produced the highest odds of achieving 5 and 10% weight loss compared to orlistat, liraglutide, lorcaserin and bupropion/naltrexone (moderate confidence in estimates) with no increased odds of adverse events (OR of 9.22 for achieving 5% weight loss relative to placebo). However, there are no data on how this drug combination influences eating behaviour, or fMRI data to provide neurophysiological correlates of behaviour. Thus, the behavioural specificity of phentermine/topiramate remains unknown and effects on the psychological and motivational aspects which underlie problematic eating behaviours have yet to be characterised. Without clear data on how the drug effects eating behaviour it is difficult to suggest which individuals may benefit from taking this drug and to assess its tailoring potential.

## A methodological platform for assessing drug action

Pharmacotherapy as an adjunct to reduced calorie diets and behaviour modification may provide additional benefit for obese people in achieving and maintaining clinically meaningful weight loss. However, so far there are little data to enable the link between pharmacology and real-world therapeutic benefits, i.e. proof of concept.

Whilst there are no mechanistic data for phentermine/topiramate, other reviewed studies provide tentative evidence that liraglutide and lorcaserin produce weight loss by reducing energy intake and increasing satiety. Other effects are less well characterised. There are self-report data for bupropion/naltrexone suggesting satiety, reward and/or IC effects; however, direct assessment of behaviour is lacking. Without appropriately designed, well-powered mechanistic studies detailing drug effects on the substrates of eating behaviour, the tailoring potential of these drugs remains speculative.

Evidence for increased IC in response to bupropion/naltrexone comes from a passive viewing fMRI paradigm. It is difficult to translate activity in singular brain regions as being a neurophysiological marker of IC without providing a behavioural correlate. Passive viewing paradigms are the only technique used in imaging experiments for pharmacotherapies and provide the sole neurophysiological markers of drug effects to base personalised treatments on. However, simple activation analyses from passive viewing paradigms are problematic for interpretation of drug effects (one can only make meaningful judgements about activity enhancement of IC regions, if the activity is in association with a task that requires an inhibitory response (see Table 3 for recommendations). If we understand appetite regulation to be an interplay between satiety, reward and IC, then using simple paradigms from the psychology literature that provide behavioural correlates of these components in combination with systems level fMRI data analysis techniques (functional connectivity, dynamic causal modelling) will provide powerful evidence to assess the mechanisms by which anti-obesity drugs work.

**Table 3:** Methodological platform for assessing drug action

	Component assessed	Specific methods	As per
*Behavioural measures*:			
Energy intake at ad libitum meals	Satiety	Measure intake at a (homogenous) lunch time meal, several hours following a fixed load breakfast (and overnight fast)	Van Can (2014) [31]—Liraglutide
Microstructure of eating and eating rate	Satiety/reward	Universal eating monitor used to measure total intake, eating rate and within-meal measures of satiety	Halford et al. (2010) [43]—Sibutramine
Food choice	Reward	Food choice (healthy/unhealthy sweet foods, healthy/unhealthy savoury foods, fatty foods) at ad libitum buffet meals can be assessed to see if drugs modify food choice for more palatable ingesta (reward driven eating). Macronutrient content of consumed food can be calculated	Martin et al. (2010) [48]—Lorcaserin
Visual probe with concurrent eye tracking	Reward	Visual probe assesses attentional bias/incentive salience of rewarding stimuli. This can be assessed with food specific cues. Using eye tracking can give an implicit measure of attentional bias to rewarding foods	Nijs et al. (2010) [6]
Cue specific inhibitory control task	Inhibitory control	Food-cue-specific inhibitory control task	Houben et al. (2014) [59]
*Subjective measures*:			
Satiety VAS	Satiety	Hunger, fullness, prospective consumption, desire to eat—100 ml VAS at hourly intervals to assess fluctuations in appetite throughout the day	Halford et al. (2010) [43] Sibutramine
Change in expected Satiety	Satiety	Food portions shown to patient who is asked to indicate how satiating they think it would be	Brunstrom et al. (2008) [60]
Satiety quotient	Satiety	Pre-meal hunger—post-meal hunger, divided by amount consumed. A measurement of satiating properties of a meal	Halford et al. (2010) [43] Sibutramine
Control of eating questionnaire	Reward/inhibitory control	COEQ—1) general food craving, 2) craving for sweet, 3) craving for savoury, 4) control over appetite	Greenway et al. (2010) [39] Bupropion/naltrexone
Power of food	Inhibitory control	A tool developed to assess effects of obesity treatments on feelings of being controlled by food	Capelleri et al. (2009) [61]
Dutch eating Behaviour questionnaire (DEBQ)	Inhibitory control	External, emotional and restrained eating patterns	De Boer et al. (2016) [62] Liraglutide
The mindful eating scale	Inhibitory control	Key environment stimuli associated with reduced control of eating	Framson et al. (2009) [63]
*Physiological and neurophysiological measures*:			
fMRI	Satiety	Activity in response to food cues in fed and fasted states	Farr et al. (2016) [32] Liraglutide 1.8 mg
fMRI—reward system	Reward	Activity and functional connectivity of reward system during receipt of palatable tastes (e.g. chocolate milk)	Van Bloemendall et al. (2014) [64] Exenetide
fMRI—inhibitory control pathway	Inhibitory control	Activity/functional connectivity analysis of brain regions active during inhibition, using food cue specific inhibitory control task	Batterink et al. (2010) [65]

In order to deliver personalised treatment with anti-obesity medication, it is necessary to characterise drug effects on phenotypic traits associated with increased energy intake. However, to date eating behaviour has not been studied in sufficient detail with current anti-obesity drugs. A methodological platform for assessment of drug effects on appetite regulation is provided in Table 3.

Clinical response to pharmacotherapy can vary greatly, as such future research with anti-obesity drugs should seek to assess sub-populations of patients who respond well to drug treatment and are successful at losing weight. Large-scale candidate gene association studies which identify genetic variants associated with weight loss response to pharmacotherapy (e.g. Li et al. [57] report genetic variation which predicts successful response to topiramate treatment) and genotyping for polymorphisms predictive of successful outcomes (e.g. Hauner et al. [58]) will help enable personalisation of pharmacotherapy for obesity. The goal of pharmacogenetics is to help identify patients who may benefit most from drug therapies and is currently underused in drug development of anti-obesity drugs. Similarly categorising sub-populations by behavioural risk factors (susceptible phenotypes) and observing which behaviour types predict successful outcomes for weight loss with each drug therapy would be beneficial for tailored pharmacotherapy. Genotyping data should be conducted in parallel with neuroimaging (and molecular imaging techniques that allow investigation of the brain–drug effect), cognitive and experimental paradigms that assess IC, food reward sensitivity and eating behaviour, to characterise drug effects on appetite and to observe predictors for successful weight loss.

## References

[1] Zhang Z, Wang M (2012). Obesity, a health burden of a global nature. Acta Pharmacol Sin.

[2] Public Health England. Making the case for tackling obesity—why invest? February 2015. http://bit.ly/1EA6iXF

[3] Blundell JE, King NA (1996). Overconsumption as a cause of weight gain: behavioural–physiological interactions in the control of food intake (appetite). Ciba foundation symposium 201—the origins and consequences of obesity, Wiley Online Library 10.1002/9780470514962.ch9 9017279

[4] Blundell JE, Cooling J (2000). Routes to obesity: phenotypes, food choices and activity. Br J Nutr.

[5] Blundell JE, Stubs RJ, Golding C (2005). Resistance and susceptibility to weight gain: individual variability in response to a high-fat diet. Physiol Behav.

[6] Nijs IM, Muris P, Euser AS, Franken IHA (2010). Differences in attention to food and food intake between overweight/obese and normal-weight females under conditions of hunger and satiety. Appetite.

[7] Massey A, Hill AJ (2012). Dieting and food craving. A descriptive, quasi-prospective study. Appetite.

[8] Warren C, Cooper PJ (1988). Psychological effects of dieting. Br J Clin Psychol.

[9] Keys A, Brožek J, Henschel A, Mickelsen O, Taylor HL (1950). The biology of human starvation.(2 vols)

[10] Jasinska AJ, Yasuda M, Burant CF (2012). Impulsivity and inhibitory control deficits are associated with unhealthy eating in young adults. Appetite.

[11] Locke AE, Kahali B, Berndt SI (2015). Genetic studies of body mass index yield new insights for obesity biology. Nature.

[12] Frayling TM, Timpson NJ, Weedon MN (2007). A common variant in the FTO gene is associated with body mass index and predisposes to childhood and adult obesity. Science.

[13] Wardle J, Carnell S, Haworth CMA, Farooqi IS, O’Rahilly S, Plomin R (2008). Obesity associated genetic variation in FTO is associated with diminished satiety. J Clin Endocrinol Metab.

[14] Llewellyn CH, Trzaskowski M, van Jaarsveld CHM, Plomin R, Wardle J (2014). Satiety mechanisms in genetic risk of obesity. JAMA pediatr.

[15] Cecil JE, Tavendale R, Watt P, Hetherington MM, Palmer CNA (2008). An obesity-associated FTO gene variant and increased energy intake in children. N Engl J Med.

[16] Wardle J, Llewellyn CH, Sanderson S, Plomin R (2009). The FTO gene and measured food intake in children. Int J Obes.

[17] Tanofsky-Kraff M, Han JC, Anandalingam K (2009). The FTO gene rs9939609 obesity-risk allele and loss of control over eating. Am J Clin Nutr.

[18] van Jaarsveld CH, Boniface D, Llewellyn CH, Wardle J (2014). Appetite and growth: a longitudinal sibling analysis. JAMA pediatr.

[19] Syrad, H, Llewellyn CH, Johnson L et al. (2016) Meal size is a critical driver of weight gain in early childhood. Scientific Reports 6 10.1038/srep28368 PMC4913249 27321917

[20] Welbourn R, Dixon J, Barth JH (2016). NICE-accredited commissioning guidance for weight assessment and management clinics: a model for a specialist multidisciplinary team approach for people with severe obesity. Obes Surg.

[21] Cabou C, Burcelin R (2011). GLP-1, the gut-brain, and brain-periphery axes. Rev Diabet Stud.

[22] Katsurada K, Yada T (2016). Neural effects of gut-and brain-derived glucagon-like peptide-1 and its receptor agonist. J Diabetes Investig.

[23] Krieger JP, Arnold M, Pettersen KG, Lossel P, Langhans W, Lee SJ (2016). Knockdown of GLP-1 receptors in vagal afferents affects normal food intake and glycemia. Diabetes.

[24] Kanoski SE, Rupprecht LE, Fortin SM, De Jonghe BC, Hayes MR (2012). The role of nausea in food intake and body weight suppression by peripheral GLP-1 receptor agonists, exendin-4 and liraglutide. Neuropharmacology.

[25] Secher A, Jelsing J, Baquero AF (2014). The arcuate nucleus mediates GLP-1 receptor agonist liraglutide-dependent weight loss. J Clin Investig.

[26] Alhadeff AL, Rupprecht LE, Hayes MR (2011). GLP-1 neurons in the nucleus of the solitary tract project directly to the ventral tegmental area and nucleus accumbens to control for food intake. Endocrinology.

[27] Khera R, Murad MH, Chandar AK (2016). Association of pharmacological treatments for obesity with weight loss and adverse events: a systematic review and meta-analysis. JAMA.

[28] Inoue K, Maeda N, Kashine S (2011). Short-term effects of liraglutide on visceral fat adiposity, appetite, and food preference: a pilot study of obese Japanese patients with type 2 diabetes. Cardiovasc Diabetol.

[29] Horowitz M, Flint A, Jones KL (2012). Effect of the once-daily human GLP-1 analogue liraglutide on appetite, energy intake, energy expenditure and gastric emptying in type 2 diabetes. Diabetes Res Clin Pract.

[30] Flint A, Kapitza C, Zdravkovic M (2013). The once-daily human GLP-1 analogue liraglutide impacts appetite and energy intake in patients with type 2 diabetes after short-term treatment. Diabetes Obes Metab.

[31] van Can J, Sloth B, Jensen CB, Flint A, Blaak EE, Saris WHM (2014). Effects of the once-daily GLP-1 analog liraglutide on gastric emptying, glycemic parameters, appetite and energy metabolism in obese, non-diabetic adults. Int J Obes.

[32] Farr OM, Sofopoulos M, Tsoukas MA (2016). GLP-1 receptors exist in the parietal cortex, hypothalamus and medulla of human brains and the GLP-1 analogue liraglutide alters brain activity related to highly desirable food cues in individuals with diabetes: a crossover, randomised, placebo-controlled trial. Diabetologia.

[33] Button KS, Ioannidis JPA, Mokrysz C (2013). Power failure: why small sample size undermines the reliability of neuroscience. Nat Rev Neurosci.

[34] Goldstein MG (1998). Bupropion sustained release and smoking cessation. J Clin Psychiatr.

[35] Anderson JW, Greenway FL, Fujioka K, Gadde KM, McKenney J, O’neil PM (2002). Bupropion SR enhances weight loss: a 48-week double-blind placebo-controlled trial. Obes Res.

[36] Caixas A, Albert L, Capel I, Rigla M (2014). Naltrexone sustained-release/bupropion sustained-release for the management of obesity: review of the data to date. Drug Des Dev Ther.

[37] Lee MW, Fujioka K (2009). Naltrexone for the treatment of obesity: review and update. Expert Opin Pharmacother.

[38] Greenway FL, Whitehouse MJ, Guttadauria M (2009). Rational design of a combination medication for the treatment of obesity. Obesity.

[39] Greenway FL, Fujioka K, Plodkowski RA (2010). Effect of naltrexone plus bupropion on weight loss in overweight and obese adults (COR-I): a multicentre, randomised, double-blind, placebo-controlled, phase 3 trial. Lancet.

[40] Apovian CM, Aronne L, Rubino D (2013). A randomized, phase 3 trial of naltrexone SR/bupropion SR on weight and obesity-related risk factors (COR-II). Obesity.

[41] Wadden TA, Foreyt JP, Foster JD (2011). Weight loss with naltrexone SR/bupropion SR combination therapy as an adjunct to behavior modification: the COR-BMOD trial. Obesity.

[42] Wang GJ, Tomasi D, Volkow MD (2014). Effect of combined naltrexone and bupropion therapy on the brain’s reactivity to food cues. Int J Obes.

[43] Halford JCG, Boyland EJ, Blundell JE, Kirkham TC, Harrold JA (2010). Pharmacological management of appetite expression in obesity. Nat Rev Endocrinol.

[44] Heisler LK, Jobst EE, Sutton GM (2006). Serotonin reciprocally regulates melanocortin neurons to modulate food intake. Neuron.

[45] Smith S, Weissman NJ, Anderson CM (2010). Behavioral modification and lorcaserin for overweight and obesity management (bloom) study group. Multicenter, placebo-controlled trial of lorcaserin for weight management. N Engl J Med.

[46] O’neil PM, Smith SR, Weissman NJ (2012). Randomized placebo-controlled clinical trial of lorcaserin for weight loss in type 2 diabetes mellitus: the BLOOM-DM study. Obesity.

[47] Fidler MC, Sanchez M, Raether B (2011). A one-year randomized trial of lorcaserin for weight loss in obese and overweight adults: the BLOSSOM trial. J Clin Endocrinol Metab.

[48] Martin CK, Redman LM, Zhang J (2010). Lorcaserin, a 5-HT2C receptor agonist, reduces body weight by decreasing energy intake without influencing energy expenditure. J Clin Endocrinol Metab.

[49] Farr OM, Upadhyay J, Gavrieli R (2016). Lorcaserin administration decreases activation of brain centers in response to food cues and these emotion-and salience-related changes correlate with weight loss effects: a four week long randomized, placebo-controlled, double-blinded clinical trial. Diabetes.

[50] Allison DB, Gadde KM, Garvey WT (2012). Controlled-release phentermine/topiramate in severely obese adults: a randomized controlled trial (EQUIP). Obesity.

[51] Rothman RB, Baumann MH, Dersch CM (2001). Amphetamine-type central nervous system stimulants release norepinephrine more potently than they release dopamine and serotonin. Synapse.

[52] Li Z, Maglione M, Tu W (2005). Meta-analysis: pharmacologic treatment of obesity. Ann Intern Med.

[53] Hendricks EJ, Greenway FL, Westman EC, Gupta AK (2011). Blood pressure and heart rate effects, weight loss and maintenance during long-term phentermine pharmacotherapy for obesity. Obesity.

[54] Bray GA, Hollander P, Klein S (2003). A 6-month randomized, placebo-controlled, dose-ranging trial of topiramate for weight loss in obesity. Obes Res.

[55] Wilding J, Van Gaal L, Rissanen A, Vercruysse F, Fitchet M (2004). A randomized double-blind placebo-controlled study of the long-term efficacy and safety of topiramate in the treatment of obese subjects. Int J Obes.

[56] Gadde KM, Allison DB, Ryan DH (2011). Effects of low-dose, controlled-release, phentermine plus topiramate combination on weight and associated comorbidities in overweight and obese adults (CONQUER): a randomised, placebo-controlled, phase 3 trial. Lancet.

[57] Li QS, Lenhard JM, Zhan Y (2016). A candidate-gene association study of topiramate-induced weight loss in obese patients with and without type 2 diabetes mellitus. Pharmacogenet Genom.

[58] Hauner H, Meier M, Jöckel K-H, Frey UH, Siffert W (2003). Prediction of successful weight reduction under sibutramine therapy through genotyping of the G-protein β3 subunit gene (GNB3) C825T polymorphism. Pharmacogenet Genom.

[59] Houben K, Nederkoorn C, Jansen A (2014). Eating on impulse: the relation between overweight and food-specific inhibitory control. Obesity.

[60] Brunstrom JM, Shakeshaft NG, Scott-Samuel NE (2008). Measuring ‘expected satiety’ in a range of common foods using a method of constant stimuli. Appetite.

[61] Cappelleri JC, Bushmakin AG, Gerber RA (2009). Evaluating the Power of Food Scale in obese subjects and a general sample of individuals: development and measurement properties. Int J Obes.

[62] de Boer SA, Lefrandt JD, Petersen JF, Boersma HH, Mulder DJ, Hoogenberg K (2016). The effects of GLP-1 analogues in obese, insulin-using type 2 diabetes in relation to eating behaviour. Int J Clin Pharm.

[63] Framson C, Kristal AR, Schenk JM, Littman AJ, Zeliadt S, Benitez D (2009). Development and validation of the mindful eating questionnaire. J Am Diet Assoc.

[64] van Bloemendaal L, IJzerman RG, Jennifer S (2014). GLP-1 receptor activation modulates appetite-and reward-related brain areas in humans. Diabetes.

[65] Batterink L, Yokum S, Stice E (2010). Body mass correlates inversely with inhibitory control in response to food among adolescent girls: an fMRI study. Neuroimage.

